# Development and validation of an *Onchocerca ochengi* microfilarial hamster model for onchocerciasis drug screens

**DOI:** 10.1186/s12879-016-1753-2

**Published:** 2016-08-11

**Authors:** Glory Enjong Mbah, Rene Bilingwe Ayiseh, Fidelis Cho-Ngwa

**Affiliations:** Department of Biochemistry and Molecular Biology, Faculty of Science, University of Buea, Buea, South West Region Cameroon

**Keywords:** Onchocerciasis, Hamsters, *Onchocerca ochengi*, Drug screens, *Loa loa*, Microfilariae, Ivermectin

## Abstract

**Background:**

Onchocerciasis, caused by the parasitic nematode, *Onchocerca volvulus* afflicts some 37 million people worldwide, and is the second leading infectious cause of blindness globally. The only currently recommended drug for treatment of the disease, ivermectin, is only microfilaricidal and has serious adverse effects in individuals co-infected with high loads of *Loa loa* microfilariae (mf), prompting the search for new and better drugs. Onchocerciasis drug discovery studies have so far been based on in vivo models using *Onchocerca* species which are not the closest to *O. volvulus,* and which may therefore, not adequately mimic the natural infection in humans. Therefore, this study was carried out to develop a better drug screening model for onchocerciasis, based on the use of cow-derived *O. ochengi*, the closest known relative of *O. volvulus*.

**Methods:**

Mf of *O. ochengi* were injected subcutaneously at the nape of Syrian hamsters (*Mesocricetus auratus*) and BALB/c mice. The skin, and especially the earlobes of the animals were examined for mf 15–31 days after infection. For selected model validation, the hamsters were treated with ivermectin at 150 or 600 μg/kg body weight and examined 30 days after infection for mf. For *L. loa* studies in hamsters, isolated mf were injected intraperitoneally and animal organs were examined on day 26 for mf.

**Results:**

The Syrian hamsters were found to be the more permissive to *O. ochengi* mf as fully viable mf were recovered from them on day 30, compared to BALB/c mice where such mf were recovered on day 15, but not 30. However, both animals were not permissive to *L. loa* mf even by day 15. Interestingly, more than 50 % of the total *O. ochengi* mf recovered were from the earlobes. The number of mf injected was directly proportional to the number recovered. Ivermectin at both concentrations tested completely eliminated the *O. ochengi* mf from the hamsters.

**Conclusion:**

This study reveals the Syrian hamster as an appropriate small animal model for screening of novel compounds against *O. ochengi*, the closest known relative of *O. volvulus*.

## Background

Onchocerciasis is a neglected tropical disease caused by the filarial nematode, *Onchocerca volvulus*. It afflicts some 37 million people globally, with 99 % of all the cases living in sub-Saharan Africa [1]. Onchocerciasis was estimated to account for 18.3 million disability-adjusted life years (DALYs) in sub-Saharan Africa within 20 years [2]. It is the second leading infectious cause of blindness worldwide [3]. The debilitating symptoms of onchocerciasis are attributed to inflammatory reactions to dead or dying *O. volvulus* microfilariae (mf) in the skin and eyes [4, 5]. The mf of *O. volvulus* is the stage of the parasite ingested during a blood meal on humans by the black fly (of genus, *Simulium*), in which the mf develop to the infective third stage larvae (L3), which may be transmitted to another person when bitten by the infected fly. Ivermectin, a microfilaricide is the only recommended drug used in many onchocerciasis elimination programs to break the transmission cycle by killing the *O. volvulus* mf. This drug is unfortunately, only a microfilaricide, and a major challenge with ivermectin treatment is the fact that it also kills *L. loa* mf in blood, a situation that often leads to severe adverse events (encephalopathy and death) in individuals with high *L. loa* mf load [6]. In addition, there is evidence of resistance or low response rate of mf to ivermectin [7, 8], emphasizing the need for new therapeutic options.

Efficacious small animal models in onchocerciasis for in vivo screens and preclinical studies, capable of closely mimicking the natural infection in humans are an urgent, unmet need. Immunocompetent rodents are non-permissive to natural infections of *O. volvulus* [9], but experimentally injected *O. lienalis* mf have been shown to survive in mice, especially in inbred CBA mice [10, 11]. The severe-combined immunodeficient (SCID) mice have been exploited as a model for onchocerciasis and are permissive to mf of *O. lienalis* [12]. The *O. lienalis* mf mouse model has been validated with ivermectin and other filaricides [10, 13]. However, *O. ochengi* in cattle is the closest in phylogeny to *O. volvulus* [14] and many onchocerciasis drug tests in vitro [15–18] and in naturally infected cows [19–21] have been done based on *O. ochengi*. However, the cow is too big, requires huge quantities of drugs, thereby rendering it too expensive for routine screening. Consequently, most funding bodies are reticent in considering the cow as a useful model for onchocerciasis. Both *O. ochengi* and *O. volvulus* are transmitted by the same black fly vectors of genus, *Simulium. O. ochengi* is confined to Africa and combines many important features of the human infection with *O. volvulus* [21]. *O. ochengi* also forms nodules with close resemblance to those of *O. volvulus* [22]*,* which can be enumerated by palpation in situ or removed for analysis during immunological or chemotherapeutic studies. Moreover, both parasites are equally susceptible to the filaricides, ivermectin and suramin [23], and tetracyclines [24]. Molecular and biochemical comparisons reveal almost identical antigenic and genomic profiles [25]. Given that *O. ochengi* is readily available and can be obtained at relatively low cost, it is currently regarded as a more practical model in onchocerciasis drug screens [26]. However, no small laboratory animal has been reported as a model to screen drugs on *O. ochengi* mf. This study therefore, was aimed at developing and validating such a model for onchocerciasis based on the use of *O. ochengi*, and verifying the usefulness of the model for *L. loa* mf counter screens.

## Methods

### Animals

Male and female Syrian hamsters (*Mesocricetus auratus*) were purchased from Charles River (France) and flown to Cameroon. These animals together with our home-bred BALB/c mice were maintained in a conventional animal house at the Biotechnology Unit of the University of Buea. Animals were given food and water *ad libidum*. Animals were aged 10–16 weeks at the time of use in the experiments. This in vivo study was reported in accordance with the ARRIVE Guidelines for reporting animal research.

### *L. loa* patients and parasites

Due to the adverse effects observed during ivermectin treatment of individuals co-infected with *Onchocerca* and high loads of *L. loa* mf [6], we questioned if the hamster and BALB/c mice were also permissive to *L. loa* mf, thereby helping to find safer therapeutic options. *L. loa* endemic areas of Edea and Mfou health districts of the Littoral and Centre regions of Cameroon, respectively, were targeted. Participants included those who consented to participate in the study as *L. loa* parasite donors after proper understanding of the intended study. Individuals eligible for participation were adults of both sexes, >21 years of age, in good health without any clinical condition requiring long term treatment. Exclusion criteria included an mf load of <5000/ml of blood and use of anti-filarial therapy. A maximum of 20 ml of venous blood was collected from eligible individuals at most 24 h before *L. loa* mf isolation.

### Extraction of mf

#### O. ochengi mf isolation

This was done following a method we described previously [15], with slight modifications. Briefly, fresh pieces of nodule-containing umbilical cattle skin were purchased from a local slaughter house, washed repeatedly with tap water until all dirt was removed, and rinsed with distilled water. The skin was towel-dried and sterilized with 70 % ethanol, allowed to dry in a laminar flow hood and firmly attached to an autoclaved cylindrical-shaped wooden block. Using sharp razor blades, criss-cross cuts were made into the skin and submerged in ICM (incomplete culture medium: RPMI-1640 containing penicillin, streptomycin and amphotericin B) for 4 h, after which, the medium was centrifuged at 2500 rpm for 20 mins using an Eppendorf 5810R centrifuge (Eppendorf, Germany) to concentrate the mf. After viability check, the number of mf were counted and adjusted to the required number for experimental infection (5000–25,000) in 200–1000 μl of ICM.

#### *L. loa* mf isolation

1 g of medium-grain Sephadex G-50 (Pharmacia Biotech, Uppsala, Sweden) was weighed and allowed to swell overnight in 20 ml of distilled water at 4 °C. Using 25 ml serological pipette as column, the swollen gel (after attaining room temperature) was transferred and allowed to settle to a bed volume of 11 ml. This column was equilibrated with 2 bed volumes of ICM, which was also the elution buffer.

The number of *L. loa* mf per ml of blood was determined microscopically. Then 1 ml of the infected blood was loaded on the column and allowed to just completely enter the gel bed before elution began. The red coloration due to red blood cells was allowed to flow through the gel completely before fraction collection started. A total of 30 ml of eluate was collected for every ml of blood loaded. Fractions were centrifuged at 2500 rpm for 20 mins using the Eppendorf 5810R centrifuge, supernatants discarded, pellets re-suspended in ICM, pooled and centrifuged as before. Sediments obtained after the second centrifugation were re-suspended in ICM and washed on 20 % percoll at 1000 rpm for 5 mins, thrice using the same centrifuge. After the third wash, the procedure was repeated twice with 1x PBS (phosphate buffered saline) in place of percoll. Sediments were finally re-suspended in a small volume of ICM, and *L. loa* mf counted using a microscope.

### Experimental infection with *O. ochengi* mf

Isolated mf were administered subcutaneously (sc) at 7250-44,100 per animal, in 1 ml of ICM using 29.5G needles at the region behind the ears (nape). Mf were 100 % viable at time of injection.

### Experimental infection with *L. loa* mf

Four hamsters were intraperitoneally infected, each with 30,000 *L. loa* mf at a viability of 98.97 % in 2 ml of PBS using syringes with 29.5G needles. Animals were sacrificed 26 days post-infection and examined for viable mf.

### Drug administration

Ivermectin (Mectizan™; Sigma Aldrich, Germany) was administered to two groups of hamsters (randomly chosen after infection) per os*,* 3 days post-infection. One group of 5 animals received ivermectin at 150 μg/kg body weight and the other at 600 μg/kg body weight, while control animals received the vehicle (1 % DMSO) only.

### Checking animals for presence of mf

#### *O. ochengi* mf

For *O. ochengi* mf recovery the skin, approximately 0.5 cm around the ears was completely shaved. Each ear was carefully cut off, rinsed in distilled water, towel-dried and sterilized with 70 % ethanol. Each pair of ears was carefully chopped up into tiny bits and incubated in 5 ml of ICM at 37 °C for 4 h under sterile conditions. During preliminary tests, the entire animal skin was shaved and the skin of other body parts, including the fore limbs, hind limbs, head, tail and trunk were chopped into tiny pieces and also examined for mf. After incubation, the total number of mf was obtained by spreading five 100 μl portions of ICM from each well on petri dishes and observing microscopically to count the mf.

#### *L. loa* mf

For *L. loa* mf recovery, 200 μl of freshly collected blood obtained by heart puncture of the animal post sedation, were thinly spread on petri dishes in 20 μl portions and observed microscopically.

After blood was collected, each animal was sacrificed by cranial dislocation and its peritoneum washed with 50 ml PBS. After microscopic observation, the peritoneal wash obtained was centrifuged at 400 × g for 15 mins, supernatant discarded and pellets resuspended. All mf in 500 μl of the resuspended pellets were counted in 100 μl sub-portions, for each animal. The animal organs, including the heart, lungs, liver, spleen, kidneys, intestines, stomach and trunk were removed and chopped into tiny pieces and each set incubated in 7 ml of PBS (pH 7.4), at 37 °C for 1 h. After incubation as for the ear lobes, portions of the chopped organs in buffer were observed microscopically for the presence of *L. loa* mf.

### Data analysis

Data were analysed using GraphPad Prism 5.0 software and statistical analyses were performed using the Kruskal-Wallis test and Dunn’s post-test. Data presented show mean ± SEM. Data were considered to show statistical significant difference when *p* < 0.05.

## Results

### The hamster is permissive to mf of *O. ochengi,* but not to those of *L. loa*

To test if the Syrian hamster was permissive to *O. ochengi* mf, three preliminary exploratory experiments were done with different numbers of mf injected in each animal (Table 1). Following exploratory testing of different numbers of mf, the number was finally harmonized to 25,000 mf/animal, unless otherwise stated. In these studies, the hamsters were able to retain *O. ochengi* mf in the skin detected on day 21 and 30 when the animals were sacrificed. Most of the *O. ochengi* mf were found in the extremities (ears, head, fore limbs and hind limbs) and less or no mf in the tail. Interestingly, more than 50 % of the total *O. ochengi* mf recovered were from the earlobes (Table 1). Preliminary experiments were also done on BALB/c mice to verify their ability to host *O. ochengi* mf for 30 days. BALB/c mice tolerated *O. ochengi* mf injected into them for 15 days, but not 30 days, as no mf was recovered on day 30. None of the animals analyzed showed any tissue or whole body pathology (Table 1). From these preliminary experiments, the hamster was considered more permissive to *O. ochengi* mf (day 30 data) and therefore, was used for the rest of the study. In addition, since the earlobes had more than 50 % of the total *O. ochengi* mf recovered, they were the only sites analyzed for the rest of this study. Unfortunately, after infecting 4 hamsters and 5 BALB/c mice, no *L. loa* mf was recovered from any of the animals (results not shown).

**Table 1:** The Syrian hamster is more permissive to *O. ochengi* mf than BALB/c mice

Animal	Sex (M/F)	Amount of *O. ochengi* mf injected	Percentage viability of mf at time of injection	Duration between mf injection and sacrifice (days)	N^o^ of mf in fore limbs	N^o^ of mf in hind limbs	N^o^ of mf in ear lobes	N^o^ of mf in tail	N^o^ of mf in head	N^o^ of mf in the trunk	Total mf recovered (% recovered)	% mf recovered in ear lobes relative to total recovery	Tissue and whole body pathology
Hamster 1	M	12,558	100 %	21	7	7	60	0	7	0	81 (0.65)	74.07	None observed
Hamster 2	M	44,100	88 %	21	125	100	600	67	117	130	1039 (2.36)	57.75	None observed
Hamster 3	M	25,000	100 %	30	27	7	353	0	67	53	507 (2)	69.63	None observed
BALB/c 1	M	25,000	100 %	15	120	20	390	20	610	180	1340 (5.36)	29.10	None observed
BALB/c 2	M	25,000	100 %	30	0	0	0	0	0	0	0 (0)	0	None observed
BALB/c 3	M	25,000	100 %	31	0	0	0	0	0	0	0 (0)	0	None observed
BALB/c 4	M	25,000	100 %	30	0	0	0	0	0	0	0 (0)	0	None observed
BALB/c 5	F	25,000	100 %	31	0	0	0	0	0	0	0 (0)	0	None observed

Different numbers of *O. ochengi* mf isolated from cattle skin on different days were injected subcutaneously at the nape of different animal types and the animals were sacrificed on indicated days and analyzed for *O. ochengi* mf (*n* = 3–5/group)

### *O. ochengi* mf recovery reduces with time and is linearly proportional to number administered

**Fig. 1:**
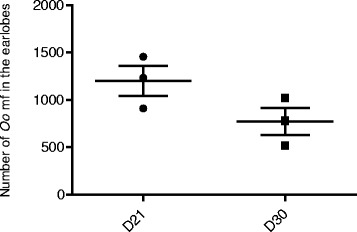
Population of *O. ochengi* in hamster decreases overtime. 20,000 *O. ochengi* mf were injected sub-cutaneously (sc) at the nape and animals were sacrificed on day 21 and 30, and mf recovered from the earlobes and counted (*n* = 3/group)

To characterize the *O. ochengi* mf hamster model, the effect of time on mf recovery post-infection was assessed. Twenty one days post-infection with 20,000 the mf, 1199 ± 158 mf were recovered from the earlobes, representing 6.0 % of the total mf injected (Fig. 1). After 30 days, 773 ± 144 *O. ochengi* mf were recovered from the earlobes, representing 3.9 % of the total mf injected. The change in mf numbers from 1199 ± 158 to 773 ± 144 in the earlobes between day 21 and 30 respectively, marks a 35.5 % drop.

**Fig. 2:**
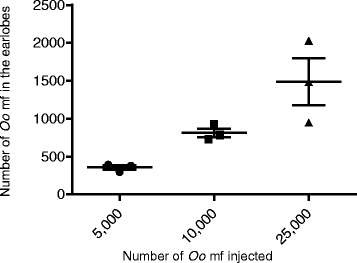
Recovery of *O. ochengi* mf in hamster is directly proportional to number of mf injected. Different numbers of *O. ochengi* mf were injected sc at the nape and animals were sacrificed on day 30 and mf recovered from the earlobes and counted (*n* = 3/group)

Next, we assessed the effect of number of *O. ochengi* mf injected on recovery. Hamsters were infected with 5000, 10,000 or 20,000 mf and their earlobes were analyzed on day 30. Interestingly, increasing the number of mf injected resulted in a linear increase in the number of mf recovered (Fig. 2). The percentage of mf recovered from the earlobes on day 30 was 7–8 %, irrespective of number of mf injected.

### Ivermectin completely clears *O. ochengi* mf from hamsters in model validation studies

**Fig. 3:**
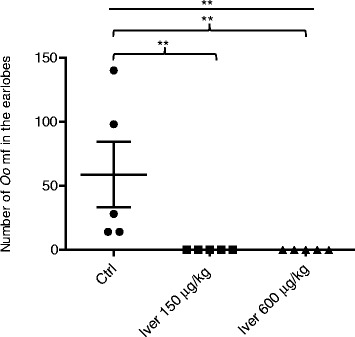
Ivermectin completely eliminates *O. ochengi* mf in hamsters. 7250 *O. ochengi* mf were injected sc at the nape per animal and animals treated once with the drug by oral gavage on day 3 at 150 or 600 μg/kg body weight. Control animals were treated with the vehicle (1 % DMSO). Animals were sacrificed on day 30 and analyzed for mf in the ear lobes (*n* = 5/group). **P < 0.01

Finally, to validate the suitability of this model, we used the drug of choice ivermectin. Three days after infection of animals with *O. ochengi* mf, 5 hamsters per group were dosed orally with ivermectin at 150 or 600 μg/kg body weight, or received the vehicle only. When animals were sacrificed on day 30, all the control animals had *O. ochengi* mf in the earlobes (Fig. 3). On the contrary, in all the animals treated with ivermectin at 150 or 600 μg/kg body weight, no *O. ochengi* mf was recovered. The control and treated groups showed a statistical significant difference (*p* = 0.0013) after a Kruskal-Wallis test. Further statistical analysis of each ivermectin-treated group versus the control group with Dunn’s post-test indicated they were significantly different (Fig. 3). These results show that the Syrian hamster is a good model to test drugs on *O. ochengi* mf.

## Discussion

The present study was carried out to develop a small immunocompetent animal model for onchocerciasis drug screens, based on the use of bovine-derived *O. ochengi*, the closest known relative of *O. volvulus*. Previously developed onchocerciasis mf models have all been based on use of mice experimentally infected with animal *Onchocerca* species which are not the closest to *O. volvulus*. We found for the first time that the Syrian hamster is a suitable model for harboring *O. ochengi* mf for more than 30 days, without any observable pathology. Although only 3 hamsters were used in preliminary studies (Table 1), the data presented in Figs. 1, 2 and 3 involving different sets of more of the animal all show that the hamster retained mf until day 30, fortifying the claim that it is a permissive host. The goal was to maintain *O. ochengi* mf for 30 days in the model, but we can say that the mf can survive longer in the hamsters, given that hundreds of mf were recovered on day 30. In our opinion, 30 days is enough time to verify reduction in mf viability, not necessarily elimination, with drug testing. None of the animals tested showed any pathology or signs of illness due to the *O. ochengi* mf infection. The model would enable preclinical drug discovery efforts in vivo to complement already well-established in vitro assays. A simple technique previously developed by our laboratory [15], permits the production of large quantities (hundreds of thousands) of *O. ochengi* mf for the screens, from an mf rich cattle skin obtainable from local slaughterhouses.

BALB/c mice were able to retain *O. ochengi* mf for 15 days. However, on day 30, no mf was recovered from all infected BALB/c mice. In contrast, all infected hamsters had many mf in almost every part of the skin, with the same or even lower number of mf injected. These observations show that the hamster is more tolerant to *O. ochengi* mf than BALB/c mice on day 30, and is therefore, a more suitable model for standard onchocerciasis drug screens. The mechanism of mf clearance by mice or other rodents is not known. From literature it is clear that the immune response plays a critical role in parasite survival in an immunocompetent host. That is why immunocompromised and transgenic animals lacking crucial cytokines like IL-4 and IL-5 have been exploited to improve filariae survival in mice. But the situation is not straightforward. For example, BALB/c mice are known to be more Th2-biased [27, 28], producing cytokines such as IL-4 and IL-5 which have been implicated in the clearing of filarial parasites from the mammalian host [29–31]. However, some filaria parasites do well in Balb/c mice than in other strains of mouse. But a fully immunocompetent host for drug screens would be better at mimicking the complex situation in humans. Our observation that the fully immunocompetent hamster is better than BALB/c mice in harboring *O. ochengi* mf for longer periods, suggest that attention should be paid to other small immunocompetent rodents (other than mice), such as gerbils in developing onchocerciasis animal models. Interestingly, other workers have shown that gerbils are permissive to *O. ochengi* adult male worms [32]. The only *O. ochengi* mouse model reported to date is the SCID mouse [32], emphasizing the need to further explore the mouse world for smaller *O. ochengi*-based models of onchocerciasis. This mouse is also permissive to adult *O. ochengi* male and female worms. However, its lack of an adaptive immune system clearly undermines the possible assisting role of an intact immune system in drug-mediated killing of parasites.

Efforts to develop a *L. loa* and *O. ochengi* mf co-infection model for drug screens were hindered by the non-permissiveness of the hamster to *L. loa* mf. More research to identify such a co-infection model would be highly beneficial to the scientific community interested in novel onchocerciasis drugs, and is currently one of the main areas of interest in our laboratory. Counter screening systems for verification of inactivity of new drugs on *L. loa* mf in vivo may help later on in avoiding or mitigating the adverse effects such as those that are observed in onchocerciasis patients co-infected with *L. loa* after ivermectin treatment [6].

As expected, the number of *O. ochengi* mf in the hamsters drops with duration of infection. The mf recovery was also directly proportional to the number administered. On day 30, the maximum recovery was 8 % which is close to the 10 % described in CBA mice 35 days after infection with *O. lienalis* [11]. The percentage of mf recovered varied between experiments, and this could probably be attributed to genetic differences between the outbred hamsters. The present model was validated with ivermectin. A dose of 150 μg/kg body weight eliminated all *O. ochengi* mf. Therefore, ivermectin will be a good positive control for testing of drugs in this model. This model can be used for drugs that are active on both micro- and macrofilariae or to acquire more data in the study of microfilaricides. It can also be used as counter screen in situations where newly developed macrofilaricide is not expected to be active against mf, to reduce adverse drug effects resulting from dying mf. However, a major limitation of the model is that it is yet to be tested or developed further to include macrofilaricidal drug screens. Further work is on-going to identify novel microfilaricides using the model and to investigate the incorporation of adult worm screens into it.

## Conclusions

This study has revealed that the Syrian hamster is a model for *O. ochengi* mf, the closest relative of *O. volvulus*. It also revealed that BALB/c mice are not permissive to *O. ochengi* mf by day 30, although they were on day 15. This is the first small laboratory animal model described for this species of mf and may serve as an important preclinical complement to in vitro and other in vivo drug screens for onchocerciasis.

## Abbreviations

DMSO, dimethyl sulfoxide; ICM, incomplete culture medium; IL, interleukin; Mf, microfilariae; O.o, *Onchocerca ochengi*; PBS, phosphate buffered saline; SCID, severe-combined immunodeficient; SEM, standard error of mean
